# Dr. Ellis W. Storms

**Published:** 1918-02

**Authors:** 


**Dr. Ellis W. Storms,** (college and date to be supplied in proof), died during the night of Jan. 8-9 at his home in Falconer. He was 50 years old and had apparently been in perfect health. Death is ascribed to cerebral haemorrhage. He had served as principal of the Ellington High School, as supervisor of the town of Cherry Creek and was for some years coroner of Chautauqua Co.

